# Mapping cover crop species in southeastern Michigan using Sentinel-2 satellite data and Google Earth Engine

**DOI:** 10.3389/frai.2023.1035502

**Published:** 2023-08-17

**Authors:** Xuewei Wang, Jennifer Blesh, Preeti Rao, Ambica Paliwal, Maanya Umashaanker, Meha Jain

**Affiliations:** School for Environment and Sustainability, University of Michigan, Ann Arbor, MI, United States

**Keywords:** cover crop, Michigan, Sentinel-2, random forest, diversified farming systems

## Abstract

Cover crops are a critical agricultural practice that can improve soil quality, enhance crop yields, and reduce nitrogen and phosphorus losses from farms. Yet there is limited understanding of the extent to which cover crops have been adopted across large spatial and temporal scales. Remote sensing offers a low-cost way to monitor cover crop adoption at the field scale and at large spatio-temporal scales. To date, most studies using satellite data have mapped the presence of cover crops, but have not identified specific cover crop species, which is important because cover crops of different plant functional types (e.g., legumes, grasses) perform different ecosystem functions. Here we use Sentinel-2 satellite data and a random forest classifier to map the cover crop species cereal rye and red clover, which represent grass and legume functional types, in the River Raisin watershed in southeastern Michigan. Our maps of agricultural landcover across this region, including the two cover crop species, had moderate to high accuracies, with an overall accuracy of 83%. Red clover and cereal rye achieved F1 scores that ranged from 0.7 to 0.77, and user's and producer's accuracies that ranged from 63.3% to 86.2%. The most common misclassification of cover crops was fallow fields with remaining crop stubble, which often looked similar because these cover crop species are typically planted within existing crop stubble, or interseeded into a grain crop. We found that red-edge bands and images from the end of April and early July were the most important for classification accuracy. Our results demonstrate the potential to map individual cover crop species using Sentinel-2 imagery, which is critical for understanding the environmental outcomes of increasing crop diversity on farms.

## 1. Introduction

Diversified farming systems, which comprise a suite of practices that reduce soil disturbance, maintain permanent soil cover, and increase crop functional diversity from plot to landscape scales (Kremen et al., 2012), have been shown to increase agricultural sustainability (Garibaldi et al., 2017; Tamburini et al., 2020; Beillouin et al., 2021). One key diversification practice is the use of cover crops, which are non-harvested crops grown in rotation with primary crops that maintain soil cover during periods that would otherwise typically be in bare fallow. A large body of evidence shows that cover crops improve multiple ecosystem functions, such as reducing soil erosion, increasing soil fertility and building soil organic carbon (C), retaining nitrogen (N) and phosphorus (P), reducing pest and disease pressure, and enhancing crop yields (Tonitto et al., 2006; Finney and Kaye, 2017; Kaye and Quemada, 2017; Blesh, 2018). Despite these potential benefits, there is little understanding of the extent to which farmers are adopting cover crops, both across space and through time. This is because existing datasets on the use of cover crops at large scales are typically collected through government censuses, which are done infrequently and produce data at the level of political boundaries. Instead, remote sensing can offer a low-cost way to simultaneously monitor cover crop adoption at the field scale and at large spatio-temporal scales.

There is a growing body of literature that has used satellite imagery to map various characteristics of cover crops, including their presence, phenology, and biomass, in multiple farming systems across the globe. For example, previous studies have used multi-spectral optical imagery, such as Landsat, and Sentinel-2, to map the presence of cover crops by detecting vegetation greenness outside of the primary growing season (Hively et al., 2015; Seifert et al., 2019; Fan et al., 2020; Thieme et al., 2020). These studies have also found that vegetation indices can be used to map the phenology, sowing time, and termination date of cover crops (Fan et al., 2020; Gao et al., 2020) as well as their performance by estimating biomass (Prabhakara et al., 2015; Breunig et al., 2020; Thieme et al., 2020; Goffart et al., 2021) and biomass nitrogen content (Xia et al., 2021). Recent work has also shown that radar and thermal data can be used in combination with optical imagery to improve cover crop area and biomass estimation (Barnes et al., 2021; Jennewein et al., 2022). These methods have been applied to map cover crops in multiple farming systems, including in the eastern United States (Hively et al., 2015), the United States Midwest (Seifert et al., 2019; KC et al., 2021), the Netherlands (Fan et al., 2020), Belgium (Goffart et al., 2021), and Brazil (Breunig et al., 2020).

While these studies have shown that the presence, phenology, and biomass of cover crops can be detected with high accuracy at the field scale, few studies have attempted to map specific cover crop species using satellite imagery. Yet knowing the composition of cover crop species is important given that the functional diversity of cropping systems—i.e., the diversity of crop functional traits—is a crucial predictor of ecosystem services, including productivity and nutrient retention (Blesh and Drinkwater, 2013; Martin and Isaac, 2015; Wood et al., 2015). It is likely that remote sensing can identify different cover crop species based on differences in phenology or spectral signatures. Considering phenology, different cover crop species may be planted or terminated at different times based on where they fall in the annual crop growing calendar. It is likely that vegetation indices that measure leaf area index and biomass, such as the Normalized Difference Vegetation Index (NDVI), can be used to map these phenological differences as NDVI will accordingly increase, peak, and decline at different times during the growing season (Jain et al., 2016; Tariq et al., 2022). In addition, it is possible that cover crops may be distinguished by differences in spectral signatures (Rao et al., 2021). For example, flowering species such as red clover, which produces purple flowers, likely have different spectral signatures than grass species, such as cereal rye, which remain green throughout the growing season.

In this study, we use Sentinel-2 satellite imagery and Google Earth Engine to map two common cover crop species, red clover and cereal rye, which represent legume and grass functional groups, in the River Raisin watershed in southeastern Michigan. We focus on this region because it has moderate levels of cover crop adoption, with ~4–8% of agricultural area under cover crops according to the latest USDA census. Furthermore, cover crop adoption in this region could have important implications for reducing soil erosion and associated N and P losses, which contribute to harmful algal blooms and eutrophication in the Great Lakes, the Mississippi Delta, and the Gulf of Mexico (Michalak et al., 2013), as well as to climate change through greenhouse gas emissions (Eagle et al., 2020; EPA, 2022). To our knowledge, the only data product that has attempted to map individual cover crop species in the United States is the Cropland Data Layer (CDL), which is produced by the United States Department of Agriculture (USDA) National Agricultural Statistics Service (NASS) using Landsat satellite data, however the accuracies of cereal rye and red clover in this data set are low (USDA, 2017). We specifically examine whether moderate-resolution optical data, Sentinel-2, can more effectively classify cereal rye and red clover in southeastern Michigan based on differences in their phenological and/or spectral signatures.

## 2. Methods

### 2.1. Study region

**Figure 1:**
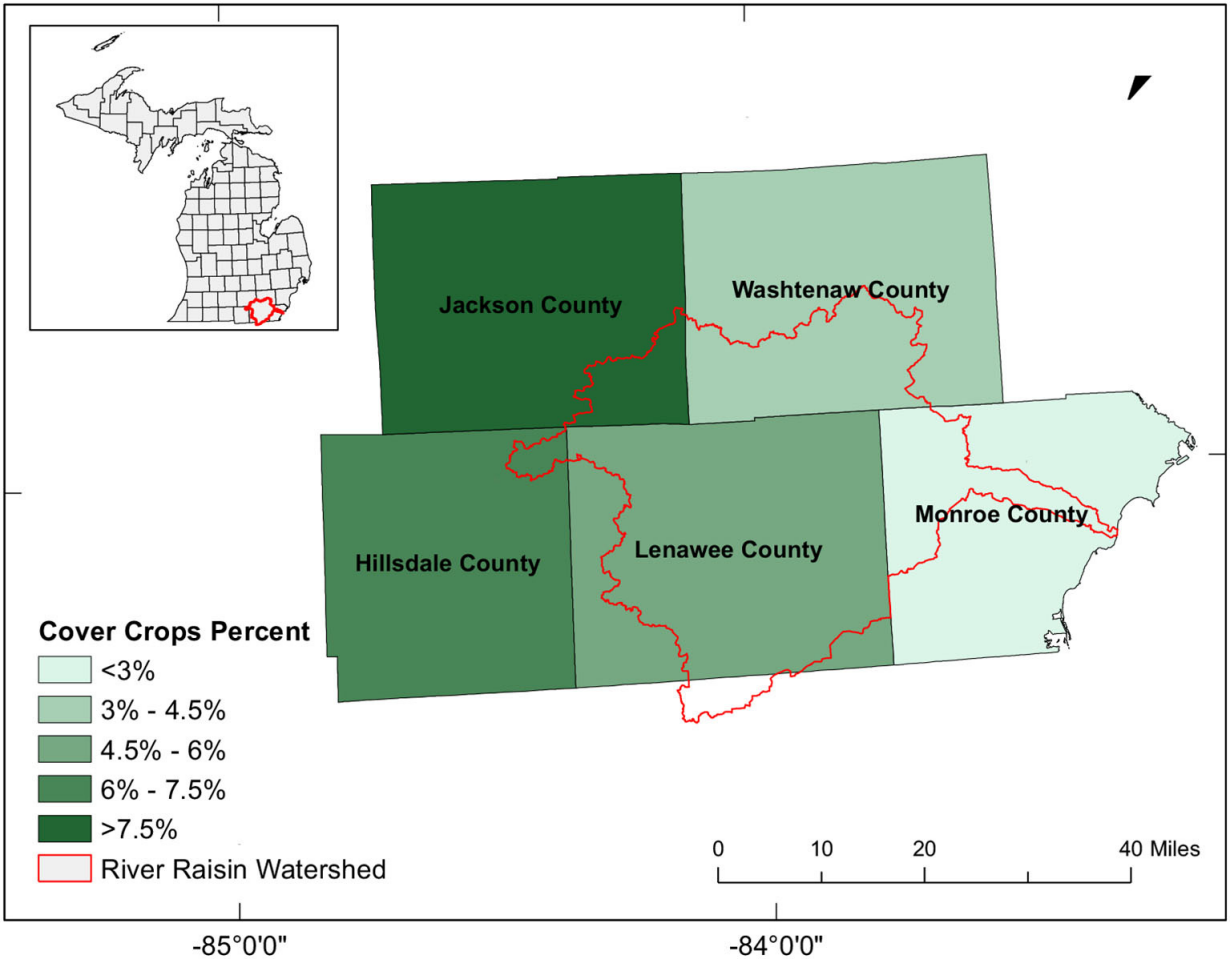
Location of the River Raisin Watershed in Michigan (highlighted in red) and the percent area under cover crops in 2017 at the county level according to the USDA Census.

Our study area, the River Raisin watershed, encompasses parts of southeastern Michigan and northern Ohio, including Washtenaw, Jackson, Lenawee, and Monroe counties (Figure 1). As a tributary to Lake Erie, water drains from the north to west and enters Lake Erie at Monroe Harbor. It is a highly productive and intensively farmed area, with over 75% of the land area under agriculture. As a result, the River Raisin watershed is a major source of N and P losses that cause eutrophication and harmful algal blooms.

**Figure 2:**
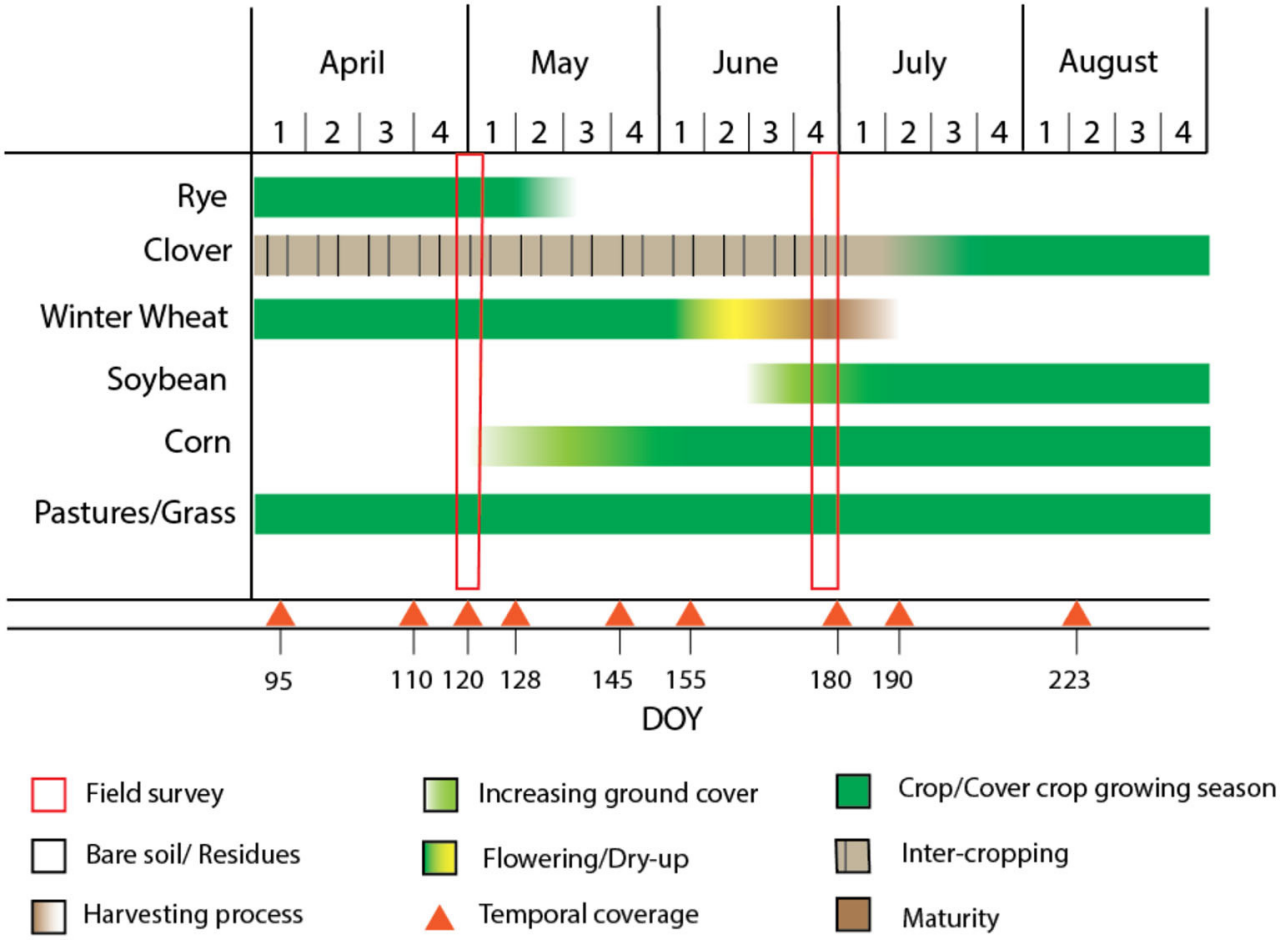
Phenology of different agricultural land cover classes found in the River Raisin watershed, with the timing of the field survey highlighted as red rectangles (Field survey in legend) and available Sentinel-2 imagery highlighted as red triangles (Temporal coverage in legend).

In this study, we focus on two cover crop species, cereal rye and red clover, which are commonly used by farmers in our study region in the overwintering niche. Cereal rye (*Secale cereale*) is a cool season annual grass and is one of the most reliable cover crops across the Midwest (Martinez-Feria et al., 2016). Cereal rye has traits that stabilize soil, retain and recycle N, P, and other nutrients, build soil C, and suppress weeds, which may increase yields over time (Snapp and Surapur, 2018). Because of cereal rye's cold tolerance, it can be planted in the late fall after harvesting corn and gain substantial biomass in the spring as the soil warms; cereal rye is typically terminated between the end of April and the middle of May ahead of planting the following crop (Pantoja et al., 2016; Figure 2). Red clover (*Trifolium pratense*) is a biennial legume cover crop species that provides moderate amounts of N through legume N fixation, positively impacting crop yield (Coombs et al., 2017; Blesh et al., 2019). In our study region, farmers often frost-seed red clover into winter wheat (i.e., in late winter, Figure 2) to improve its establishment and minimize competition with cash crops (Gibson, 2006; Gaudin et al., 2013). Red clover grows rapidly after wheat is harvested, and usually remains in the field until the following spring.

The main cash crops grown in this region are corn and soybean, with some winter wheat. Winter wheat is planted in the fall and flowers and senesces in the middle of June (Figure 2). By the first week of July, winter wheat typically reaches maturity and is ready for harvest. Soybean and corn are both typically planted in early to mid-May, and both crops grow throughout the summer. The other common vegetation class in our study area is grass and pasture for grazing, and this class of perennial species remains green throughout the summer growing season (Figure 2).

### 2.2. Ground data collection

Based on the timing of cover crop growth in our study region (Figure 2), we conducted two separate field visits: the first occurred from April 25^th^ to May 5^th^ and the second occurred from June 23^rd^ to June 25^th^ in 2018. During these field visits we collected ground truth data that we used to calibrate and validate our remote sensing algorithm. During the first field visit, we identified the presence of cover crops during a time when other cash crops (except for winter wheat) were not prevalent. During the second field visit we were better able to distinguish cereal rye cover crops from winter wheat, as these two crops looked similar at the end of April (Figure 2).

To select appropriate fields to visit for our ground survey, we conducted the following steps. First, we used a crop mask to identify all cultivated land in our study region using the USDA NASS Cropland Data Layer (USDA, 2017). This data product defines cultivated area as pixels that were classified as cultivated in at least two out of the last five years. We then selected 5,000 random points from these cultivated pixels that were within 10 meters from a road; we did this to ensure that the fields we visited were visible from the road and could be classified easily while driving. Finally, to ensure that our randomly selected ground truth points represented the range in vegetation land cover types found across our study region, we created a maximum Normalized Difference Vegetation Index (NDVI) mosaic for April 2018 using Sentinel-2 Level-1C top of the atmosphere (TOA) satellite data in Google Earth Engine (https://earthengine.google.com/, Supplementary Figure 1). We selected 500 points from our original random sample that were stratified across four intervals of NDVI (<0.2; 0.3–0.4; 0.4–0.6; and >0.6). We then visited these 500 points in person to collect observational data on the type of land cover.

For each field, three GPS locations were taken at the start, middle, and end of the field along the road, and we recorded the observed land cover type. The five classes observed were fallow fields, grass/pastures, winter wheat, cereal rye, and red clover. Since winter wheat and cereal rye were difficult to distinguish in the first field survey, we revisited all winter wheat and cereal rye fields during the second survey in late June. At this point we were able to distinguish between these two classes because cereal rye had been terminated whereas winter wheat was still in the field and was reaching the stage of flowering or senescence. Some fields were omitted from our survey as they were either not cultivated or we were unable to identify the landcover type. In total, we collected GPS and groundcover data from 461 fields, with 173 fallow, 125 wheat, 69 grass/pasture, 64 red clover, and 30 cereal rye fields.

To delineate field boundaries, we overlaid our GPS points on a high-resolution (0.3 m) base map using Digital Globe imagery in ArcGIS 10.6.1. For each set of three GPS points which represented the start, middle, and end of each field along the road, we identified each associated visible field boundary in the high-resolution imagery. We then manually drew polygon boundaries for each field based on visual interpretation of field boundaries from the high-resolution imagery. To minimize edge effects, we buffered each field by manually shifting the polygon inwards by 10 m across all edges using ArcGIS software.

### 2.3. Satellite image processing and land cover classification

**Figure 3:**
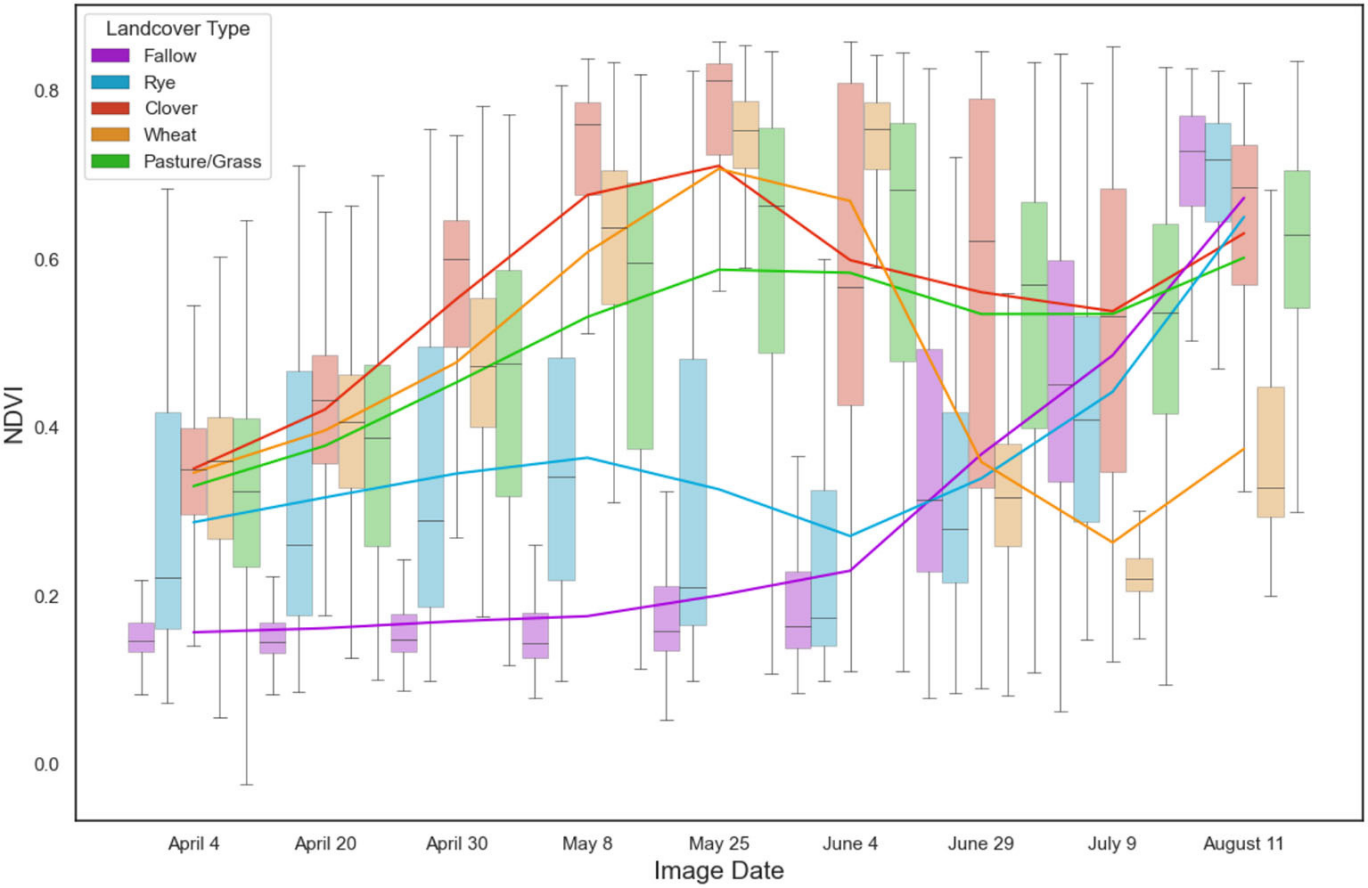
Boxplots of NDVI for each of the five land cover classes from our sample points across the nine image dates considered in our study. Trend lines for each class are drawn in colors associated with each land cover type. Considering large changes in NDVI trend lines, the sharp decrease in wheat NDVI in June represents senescence and eventual harvest of the crop, increases in NDVI in June for fallow are due to growth of the main cash crop, and increases in NDVI for rye in June represent the establishment and growth of the subsequent cash crop following the termination of rye.

We acquired Sentinel-2 Level-1C TOA satellite data from Google Earth Engine from April 4^th^ to August 11^th^, 2018, which is the key period to identify overwintering cover crops in temperate climates (Figure 2). To ensure cloud cover did not impact our analyses, we filtered images to those that had <5% cloud cover across the entire scene and visually examined each image to make sure there was no cloud cover over our study area. In total, we acquired satellite data for 11 dates throughout the growing season, with one Sentinel-2 scene covering our entire study region. Since three of these dates were within 1 week of each other (July 4 to July 9) and provided similar information, we selected only one of these image dates (July 9). This resulted in a total of 9 images throughout the growing season that we used in our analyses (Figure 3).

Previous studies show that different indices can help differentiate agricultural land cover. Here, we expected that indices that measure leaf area index, plant senescence, and leaf chlorophyll would be able to capture phenological differences across the different vegetation types considered in our study. Individual bands, such as blue, green, and red, on the other hand are more likely to capture potential spectral differences among vegetation types, such as flowering red clover and a grass such as rye. We thus calculated a suite of vegetation indices that prior research has shown to be important using band math in Google Earth Engine (Table 1). We exported all bands and indices (Table 1) for each of the nine image dates (Figure 3) for all pixels within our 461 polygons using the ExportTable function in Google Earth Engine.

**Table 1:** List of all bands and indices used in this study, along with description and references.

**Bands**		**Indices**			
**Band**	**Description**	**Index**	**Calculation**	**Description**	**References**
B2	Blue (B)	Normalized difference vegetation index (NDVI)	(NIR-R)/(NIR+R)	Leaf area index	Tucker, 1979
B3	Green (G)	Green-blue NDVI (GBNDVI)	(NIR-(G+B))/(NIR+(G+B))	Leaf area index	Wang et al., 2010
B4	Red (R)	Green-red NDVI (GRNDVI)	(NIR-(G+R))/(NIR+(G+R))	Leaf area index	Wang et al., 2007
B5	Red Edge 1 (RE1)	Red-edge normalized difference index (NDI)	(RE1 – R)/(RE1 + R)	Leaf area index	Pérez et al., 2000
B6	Red Edge 2 (RE2)	Plant senescence reflectance index (PSRI)	(R – G)/RE2	Plant senescence	Merzlyak et al., 1999
B7	Red Edge 3 (RE3)	NIR-Green NDVI (NGNDVI)	(NIR – G)/(NIR + G)	Plant senescence	Qi et al., 2002
B8	Near infrared (NIR)	Red-edge chlorophyll index (CIre)	RE3/RE1−1	Leaf chlorophyll	Gitelson et al., 2003
B8A	Red Edge 4 (RE4)	Green chlorophyll vegetation index (GCVI)	NIR/G−1	Leaf chlorophyll	Gitelson et al., 2003
B11	Shortwave infrared 1 (SWIR1)	Normalized pigment chlorophyll ratio index (NPCI)	(R – B)/(R + B)	Leaf chlorophyll	Penuelas et al., 1997
B12	Shortwave infrared 2 (SWIR2)	Shortwave infrared water stress index 1 (SIWSI1)	(NIR-SWIR1)/(NIR+SWIR1)	Vegetation moisture	Fensholt and Sandholt, 2003
		Shortwave infrared water stress index 2 (SIWSI2)	(NIR-SWIR2)/(NIR+SWIR2)	Vegetation moisture	Fensholt and Sandholt, 2003
		Normalized difference tillage index (NDTI)	(SWIR1- SWIR2)/(SWIR1 + SWIR2)	Residue cover	van Deventer et al., 1997

We imported the data into R Project software for all subsequent analyses and used random forest to classify the five land cover classes in our study region. Before running the random forest analysis, we removed highly correlated features that had a correlation value greater than 0.9 using the caret package (Kuhn, 2022) in R Project software (R Core Team, 2022). In addition, to reduce the effect of spatial autocorrelation, we selected 30 pixels at random from each polygon; for fields that were smaller than 30 pixels, we selected all available pixels within the field polygon. We then split our data into training (70% of fields) and validation data (30% of fields) to ensure that an independent dataset was used for validation. Random forest analyses were run using the randomForest package (Liaw and Wiener, 2002) in R Project Software using default values. We conducted accuracy assessment by applying our trained random forest model to the independent validation dataset, and used a contingency table to calculate user's accuracy (also known as precision), producer's accuracy (also known as recall), overall accuracy, and F1 scores (Supplementary Table 1). Finally, we calculated variable importance of our predictor variables by examining the mean decrease in model accuracy that occurred after each predictor variable was permuted from the full model using the randomForest package in R Project software.

## 3. Results

Our model had an overall accuracy of 83.01%, although classification accuracy varied across the five land cover classes considered in our model (Table 2). In particular, the classification of wheat was highly accurate, with an F1 score of 0.93, user's accuracy of 93.43%, and producer's accuracy of 92.75% (Tables 2, 3). Fallow lands also had high classification accuracy, with an F1 score of 0.87, user's accuracy of 79.37%, and producer's accuracy of 93.53% (Tables 2, 3). The relatively low user's accuracy was because many pixels classified as fallow were in fact other land cover classes, particularly grass/pasture. The two cover crop species had moderate classification accuracies, with the classification accuracy of red clover outperforming that of cereal rye. Red clover had an F1 score of 0.77, user's accuracy of 86.24%, and producer's accuracy of 69.29% (Tables 2, 3). The relatively low producer's accuracy was because red clover pixels were often misclassified, usually as fallow and grass/pasture. Cereal rye had an F1 score of 0.70, user's accuracy of 78.08%, and producer's accuracy of 63.33% (Tables 2, 3). The relatively low producer's accuracy occurred because cereal rye pixels were also often classified as other land cover types, particularly fallow fields. Finally, the class that had the lowest classification accuracy was grass/pasture, with an F1 score of.61, user's accuracy of 72.02%, and producer's accuracy of 52.16% (Tables 2, 3). The low producer's accuracy was again because many grass/pasture fields were misclassified as fallow fields.

**Table 2:** Contingency table and overall accuracy (OA), user's accuracy (UA) or precision, and producer's accuracy (PA) or recall of the classified validation dataset.

		**Predicted class**					
		**Clover**	**Fallow**	**Grass/pasture**	**Rye**	**Wheat**	**PA or recall**
Ground truth class	Clover	395	73	69	16	17	69.30%
	Fallow	3	1,504	33	14	4	96.53%
	Grass/Pasture	55	183	314	0	50	52.16%
	Rye	0	97	2	171	0	63.33%
	Wheat	5	38	18	18	1,011	92.75%
	UA or precision	86.24%	79.37%	72.02%	78.08%	93.44%	
						OA	83.01%

We shaded accuracies from lighter to darker to represent lower to higher percent accuracies, respectively.

**Table 3:** F1 scores for each of the five land cover classes considered in our study.

**Clover**	**Fallow**	**Grass/Pasture**	**Rye**	**Wheat**
0.77	0.87	0.61	0.70	0.93

**Figure 4:**
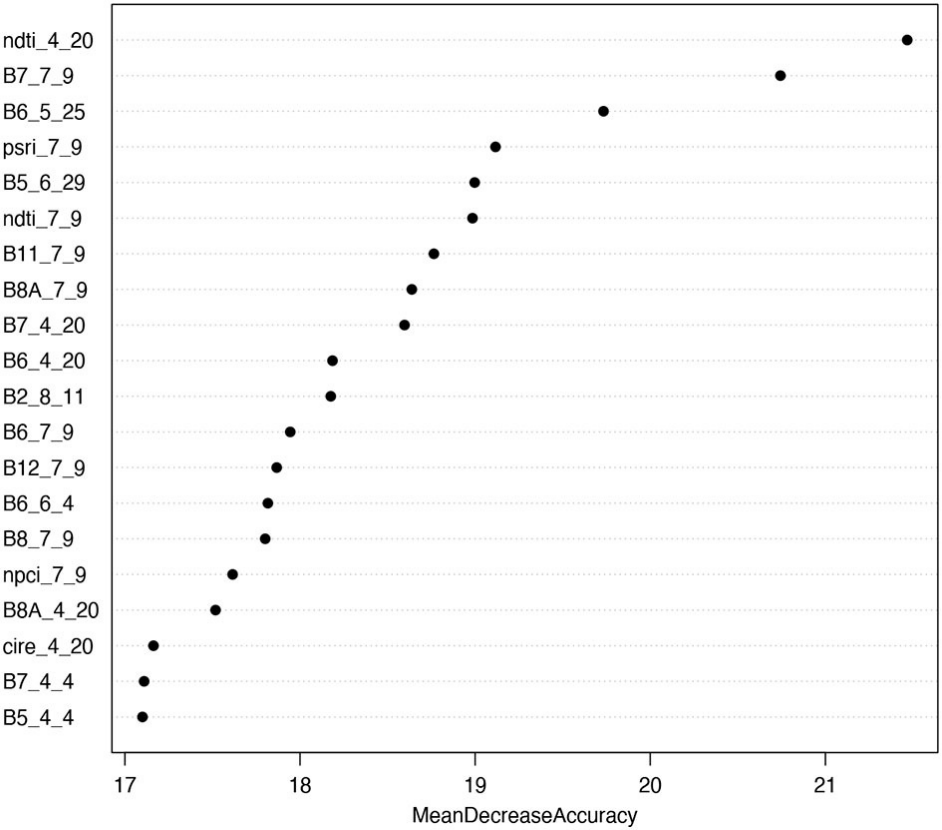
The 20 most important predictor variables in the random forest analysis considering the mean decrease in model accuracy (%) when each variable is permuted. Bands are labeled as the index or band name followed by the month and then the date of the band.

Considering variable importance, we found that the most important predictor variable for our classification was the normalized difference tillage index (NDTI) from April 20 (Figure 4). Other indices found to be within the top 20 most important variables included PSRI, NPCI, and CIRE. When examining the importance of individual raw bands, we found that the red edge and NIR bands (B5, B6, B7, B8, and B8A) were important, comprising 12 of the top 20 most important variables. Considering important time periods, we found that 9 of the 20 most important variables were bands and indices from July 9, suggesting that this is a critical time point to differentiate the different land cover classes considered in our study. In addition, 7 of the 20 most important variables were bands and indices from early in the growing season during the month of April. This suggests that this period is also critical for identifying the different land cover classes considered in our study.

## 4. Discussion and conclusion

This study used Sentinel-2 satellite data and random forest to classify two cover crops species that are grown in the overwintering period, cereal rye and red clover, in Southeast Michigan. These species represent two contrasting crop functional types (legume and grass) that can provide complementary ecosystem services in diversified cropping systems (Martin and Isaac, 2015; Wood et al., 2015), particularly N supply and nutrient retention (Tonitto et al., 2006; Blesh and Drinkwater, 2013). Overall, our model distinguished different agricultural land cover types, including these cover crops, with moderate to high accuracies. Our model reached an overall accuracy of 83%, and F1 scores for all cover and cash crops ranged from 0.7 to 0.93 (Tables 2, 3). While cover crop accuracies were moderate (user's and producer's accuracies ranged from 63.3% to 86.2%) (Table 2), they were much higher than accuracies from the existing cropland data layer (CDL) product that uses Landsat satellite data to map crop species across the United States. This suggests that locally-calibrated random forest models using Sentinel-2 data can be used to more accurately quantify the presence of individual cover crop species, which is important for better understanding their potential environmental benefits at large spatial and temporal scales.

Although overall accuracy was high, misclassifications of cover crop species were common. Red clover was most often misclassified as fallow and grass/pasture fields, likely because clover, which is a biennial species, can have biomass production similar to perennial grass/pasture fields in the summer, and may also be mowed for hay. Cereal rye was misclassified most as fallow fields, which may be due to the highly variable biomass production for cereal rye in the region based on planting date and weather conditions. Some fields with low cereal rye biomass appeared similar in spectral signature to fallow fields with weeds. Another reason for these misclassifications is that cover crops can be interseeded with a grain crop (e.g., in our study this is a common practice for red clover), or planted into remaining crop residue, which leads to substantial variation in spectral signatures across fields and even within the same field. For example, cereal rye is often planted in fields where corn was previously harvested, with the rye growing around the corn stubble. This is likely why the most common misclassification of cereal rye was with fallow fields, which often contained crop residues along with weeds. Red clover had better classification accuracies than cereal rye, likely because it has more dense ground coverage and is a broad-leaf plant, and thus was less likely to be confused with other land cover classes such as fallow fields.

Considering which bands and indices were the most important for classification, we found that the normalized difference tillage index (NDTI) from April 20 was the most important variable. This is likely because this index captures the difference between tilled and untilled fields, which helps to distinguish tilled fallow fields from the other four land cover types that remain untilled and contain vegetation (Figure 3). We also found that red-edge bands were particularly important for classifying our various land cover classes, which highlights the critical importance of using Sentinel-2 for cover crop classification. This finding supports previous studies that have found that red-edge bands can effectively classify different vegetation types, including individual crop species (Immitzer et al., 2016; Forkuor et al., 2018). Considering image timing, we found that early July (July 9^th^) followed by late April (April 20^th^-30^th^) were the most important dates for classifying the five land cover types in our study. In particular, these two time periods are critical for distinguishing the cover crop species of interest in our study region. This is because July 9^th^ is near the timing of harvest for winter wheat and high biomass growth for red clover (Figure 3), making it easier to detect fields planted with red clover during this period. Late April, on the other hand, is critical for classifying cereal rye as this is the time period when the cover crop approaches peak biomass prior to termination (Figure 3). These dates are also likely important for distinguishing other land cover classes in our study region. Specifically, on July 9 there was high biomass of cash crops that had been planted in fallow fields (Figure 3), whereas fallow fields showed little vegetation biomass during late April, especially if they had been recently tilled (Figure 3).

Our study provides some of the first evidence that Sentinel-2 can map individual cover crop species that provide critical—and distinct—ecosystem functions in the Great Lakes region. However, there are several limitations that should be addressed in future studies. First, we were unable to collect a large sample size for cover cropped fields, particularly for cereal rye (*n* = 30). This is likely because cover crops are only planted on 4–8% of agricultural land area in this region, and our random sampling approach for collecting field data did not effectively target cover cropped fields. Future work should identify if classification accuracies improve when a greater number of cover cropped fields are used to train the classifier. Second, our study was restricted to one watershed, and it is unclear how generalizable our findings would be to a larger or different study region. This is particularly true given that even within the River Raisin watershed cover crop management and performance was heterogeneous, and such heterogeneity is only expected to increase across a broader study area.

In conclusion, we find that we were able to map individual cover crop species using Sentinel-2 satellite data and random forest with moderate accuracies. This is important given that previous work has largely mapped the presence or absence of cover crops, without distinguishing species or functional types. However, given current understanding of the links between crop functional diversity and ecosystem services in agricultural systems (Martin and Isaac, 2018), better characterizing diversification through cover crops is important for linking distinct cover crop species to expected environmental benefits. Future work should examine how generalizable our findings are to broader regions in Michigan, across the Midwest, and in other cropping systems across the globe where cover crops are grown. Our results show that red-edge bands and images from early in the growing season (April), close to termination of overwintering annual cover crops, and during the summer growing season (July), were the most important for classifying agricultural land cover, including cover crops.

## Data availability statement

The raw data supporting the conclusions of this article will be made available by the authors, without undue reservation.

## Author contributions

This study was conceived of by MJ and JB. Methods were developed by MJ, JB, PR, and XW. Ground and satellite data were collected and processed by XW, MU, PR, and AP. Analyses were conducted by XW and MJ. The first draft of the manuscript was written by XW and MJ. All authors contributed to editing the manuscript.
