# Supplementary material for: Mapping cover crop species in southeastern Michigan using Sentinel-2 satellite data and Google Earth Engine
Source: Front Artif Intell. 2023 Aug 17;6:1035502. doi: 10.3389/frai.2023.1035502 (PMC10474576; doi:10.3389/frai.2023.1035502)
Supplement: Supplementary file 1 [file Data_Sheet_1.docx]

Figure S1. Maximum NDVI classes using April 2018 Sentinel-2 imagery for all cultivated pixels across our study region.

**
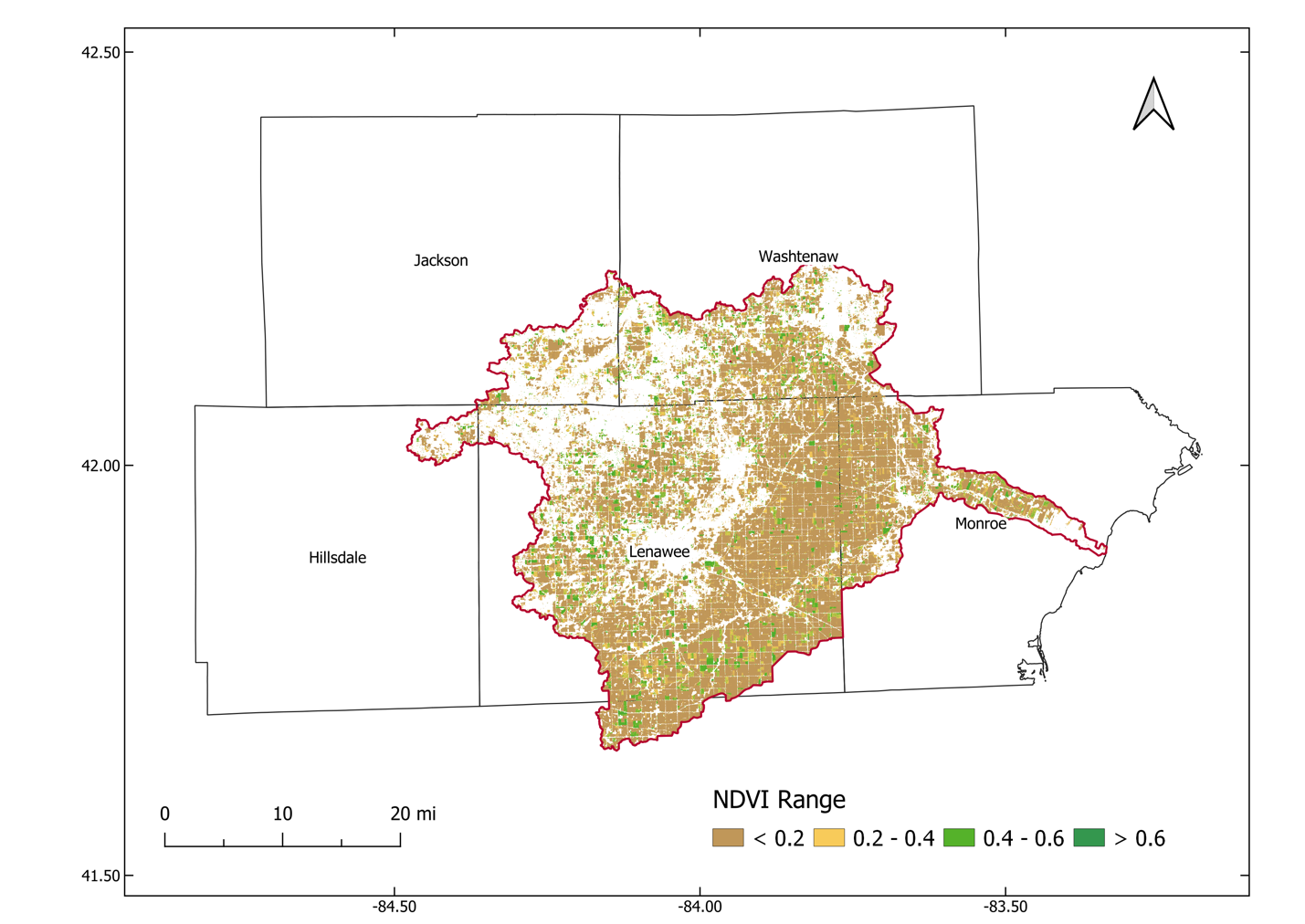
**

**Table S1.** Definitions and formulas for the four accuracy metrics used in this study.

| **Accuracy Metric** | Definition | Formula |
| --- | --- | --- |
| User’s accuracy or Recall | Probability that a feature seen on the map will actually be present on the ground | Number of correctly classified validation pixels/total number of validation pixels (calculated for each feature class) |
| Producer’s accuracy or Precision | Probability that true features on the ground are classified as such on the map | Number of correctly classified validation pixels/total number of classified pixels (calculated for each feature class) |
| Overall accuracy | Of all validation data, what proportion were mapped correctly | Number of correctly classified validation pixels/total number of validation pixels (calculated across all feature classes) |
| F1 Score | Harmonic mean of precision and recall | 2 x (Precision x Recall)/(Precision + Recall) |
